# Investigating the Sensitivity of Low-Cost Sensors in Measuring Particle Number Concentrations across Diverse Atmospheric Conditions in Greece and Spain

**DOI:** 10.3390/s23146541

**Published:** 2023-07-20

**Authors:** Georgios Kosmopoulos, Vasileios Salamalikis, Stefan Wilbert, Luis F. Zarzalejo, Natalie Hanrieder, Stylianos Karatzas, Andreas Kazantzidis

**Affiliations:** 1 Laboratory of Atmospheric Physics, Department of Physics, University of Patras, GR 26500 Patras, Greece; akaza@upatras.gr; 2 NILU—Norwegian Institute for Air Research, P.O. Box 100, 2027 Kjeller, Norway; vsal@nilu.no; 3 Institute of Solar Research, German Aerospace Center (DLR), Paseo de Almería 73, 04001 Almería, Spain; stefan.wilbert@dlr.de (S.W.); natalie.hanrieder@dlr.de (N.H.); 4 Renewable Energy Division, CIEMAT Energy Department, Avenida Complutense, 40, 28040 Madrid, Spain; lf.zarzalejo@ciemat.es; 5 Civil Engineering Department, University of Patras, GR 26500 Patras, Greece; stylianos.karatzas@outlook.com

**Keywords:** particulate matter, mass concentration, number concentration, low-cost sensors, sensors’ particle-size selectivity

## Abstract

Low-cost sensors (LCSs) for particulate matter (PM) concentrations have attracted the interest of researchers, supplementing their efforts to quantify PM in higher spatiotemporal resolution. The precision of PM mass concentration measurements from PMS 5003 sensors has been widely documented, though limited information is available regarding their size selectivity and number concentration measurement accuracy. In this work, PMS 5003 sensors, along with a Federal Referral Methods (FRM) sampler (Grimm spectrometer), were deployed across three sites with different atmospheric profiles, an urban (Germanou) and a background (UPat) site in Patras (Greece), and a semi-arid site in Almería (Spain, PSA). The LCSs particle number concentration measurements were investigated for different size bins. Findings for particles with diameter between 0.3 and 10 μm suggest that particle size significantly affected the LCSs’ response. The LCSs could accurately detect number concentrations for particles smaller than 1 μm in the urban (R^2^ = 0.9) and background sites (R^2^ = 0.92), while a modest correlation was found with the reference instrument in the semi-arid area (R^2^ = 0.69). However, their performance was rather poor (R^2^ < 0.31) for coarser aerosol fractions at all sites. Moreover, during periods when coarse particles were dominant, i.e., dust events, PMS 5003 sensors were unable to report accurate number distributions (R^2^ values < 0.47) and systematically underestimated particle number concentrations. The results indicate that several questions arise concerning the sensors’ capabilities to estimate PM_2.5_ and PM_10_ concentrations, since their size distribution did not agree with the reference instruments.

## 1. Introduction

Airborne particle measurements methods rely on high-precision reference instruments. However, their high installation and maintenance costs and bulk and heavy size impede their implementation and limit their operation to a few sites. Hence, their ability to map particulate matter (PM) gradients in high spatial and temporal resolution is limited. 

Statistical models can also be used to indirectly predict PM_2.5_ concentrations and enhance existing monitoring network resolutions. Meteorological data from a high number of weather station models in synergy with neural networks could provide real-time estimations of PM_2.5_ concentrations [1]. Moreover, Xu et al. (2016) used hidden Markov models to quantify PM mass concentration distribution in Xiamen (China) using meteorological data [2]. 

Recent advances in electronics, Internet of things (IoT), and low-cost sensing techniques offer new capabilities for continuous air quality data collection. In recent decades, numerous inexpensive PM sensors have been developed and are currently available on the market. Low-cost PM sensors (LCSs) could enable a more comprehensive understanding of PM concentration variability in fine spatial and temporal scales [3]. LCSs offer a promising tool to create large and dense air quality networks for ubiquitous PM monitoring, or even cost-effectively supplement existing regulatory networks. A dense LCS monitoring network could yield additional information and understanding of air pollution sources, especially within urban environments [4,5,6]. It is apparent that LCSs are significantly less expensive compared to the conventional instruments. Consequently, potential operation sacrifices, concerning their main components (light source, photodiode, fan, etc.), could generate-lower quality data. Moreover, LCSs’ lower and upper size detection limits often differ from those reported by the manufacturer and are rather unclear. On the other hand, the reference instruments are able to represent “real” ambient conditions reporting high-quality data. Additionally, while research-grade instruments are equipped with dryers that remove any excess water, when RH levels are increased, this is not feasible for LCSs due to their lower costs. Consequently, several questions remain on the feasibility of LCSs, regarding their long-term drift, performance, and accuracy of measurements [7]. To better understand LCSs’ capabilities, many studies have focused on the evaluation and the challenges arising from their widespread application. 

Since many questions arise regarding LCS data quality, consistent testing and calibration are crucial before deployment in operational mode. Several works and projects have described the thorough evaluation of LCSs’ PM concentration measurements in laboratory and ambient conditions [8,9]. The South Coast Air Quality Management District (SCAQMD) in the USA has also compared various particulate matter sensors against reference instruments [10]. 

A suitable LCSs calibration approach is important in order to ensure measurement accuracy [11,12]. The most common calibration technique is through colocation with Federal Referral Methods (FRM) or Federal Equivalent Methods (FEM) instruments which comply with the EN 12341 standard of the European Committee for Standardization (CEN) and the National Ambient Air Quality Standards (NAAQS) set by the U.S. Environmental Protection Agency (EPA), or additionally by using machine learning methods to develop calibration models [13,14,15,16,17]. PMS 5003 is an extensively studied sensor in various environments which shows good intra-unit correlation [18,19]. These studies underline the need to calculate and apply an appropriate correction factor that should be used on raw PMS 5003 measurements to obtain meaningful data [20,21,22,23,24].

PMS 5003 are optical counters that do not directly measure PM concentrations [8]. Their operation exploits scattering principles to calculate particle numbers and categorize particles into different size classes. PM mass concentrations are calculated using a proprietary algorithm that converts number concentration (NC) measurements to mass concentrations [9]. Several challenges arise regarding the limit of detection (LoD) of LCSs. Small (0.1–0.3 μm) and coarser (>2.5 μm) particle measurement precision is in dispute due to weak light scattering or inability to sufficiently draw large particles into the sampling chamber due to inertial deposition losses along the sampling path [9,21]. Instead of mass concentration, a more straightforward evaluation of their size selectivity and calibration of the number of particle counts could be more effective in providing more information on their operation protocols. 

However, few studies have investigated such sensors’ particle-size detection efficiency range. Kuula et al. [25] investigated the particle size response of six commercial LCSs under laboratory conditions. The results suggest that the sensor’s ability to characterize particle sizes is narrower than the manufacturer’s specification. Additionally, they reported that PMS 5003 sensors could not be used for coarse-mode particle (2.5–10 μm) measurements. The particle size selectivity conditions of PMS 5003 sensors were also investigated under controlled conditions [26]. The sensor was exposed to particles with varying properties (size, composition, and concentration) and the results suggested a relatively low ability to distinguish different particle size distributions. PMS 5003 sensors reported similar distributions for all the examined size bins, leading to ambiguous particle size classification. In addition, it was reported that particles outside the detection limit of the size channels were found to contribute to the sensor outputs. Tryner et al. [27] reported similar results. PMS 5003 bin counts differed from the actual particle distribution of the reference equipment for various aerosol types. Moreover, the measured number size distribution was rather similar for particles of varying size and composition. 

The reliability of PMS 5003 sensors to accurately capture particle NC was tested by Quimette et al. [28], indicating that the six size fractions reported do not accurately represent the particle size distribution. Moreover, it was suggested that sensor behavior was more similar to a nephelometer rather than an optical counter. The particle size effect on sensor performance was also reported during experiments conducted in field and laboratory environments [23,29,30,31]. When exposed to monodisperse particles with varying sizes (100–700 nm), PMS 5003 sensors could not accurately detect and apportion particles into correct size bins.

Despite all of the challenges and limitations reported above, several methods and tools can improve sensor size distribution selectivity. Applying a different calibration factor for each size bin improved sensor performance [9]. Moreover, Zou et al. [30] proposed that source-specific calibration factors could improve PMS 5003 sensor readings. Proper calibration of particle size distribution measurements also facilitated the PM mass concentrations reported by PMS sensors. Wallace et al. [32] exploited the raw particle NC measurements given by PMS 5003 sensors to calculate PM_2.5_ concentrations. This method’s results outperformed the PM data reported by the sensors.

The main objective of this study was to investigate LCSs capabilities in terms of particle NC measurements. Since LCS performance is location-specific, three areas (e.g., a background site and an urban site in Patras, Greece, and a semi-arid area in Almería, Spain) with distinct characteristics regarding the airborne particle sizes and the general atmospheric conditions were examined. For intercomparison purposes, the low-cost units were collocated with reference instruments.

This work is structured as follows. Section 2 describes the instrumentation, the study areas and the data treatment procedure. Section 3 presents the results and the performance of LCSs under different atmospheric conditions. Finally, Section 4 reports the main outcomes and possible future directions.

## 2. Data Collection

### 2.1. Low-Cost Sensors

The evaluation of the LCSs’ size distribution selectivity included the three (3) LCS units to be collocated next to a FEM monitor in 3 sites with different characteristics. The discrepancies among the site’s atmospheric conditions could provide more comprehensive insights into how particle sources, composition, and size affect the sensor’s behavior. In this study, the examined commercial LCSs contained two identical particle sensors (PMS 5003), namely sensor A and sensor B, that output data of PM mass concentration as well as particle NC. A pair of LCSs at each module is beneficial to assure the consistency of the measurements. Highly correlated data among the two intergraded LCSs indicates the unit’s reproducibility and precision.

According to the manufacturer, the laser scattering particle sensors have a detectable size range of 0.3–10 μm and output data that refer to the particle size distribution in six size bins; namely >0.3 μm, >0.5 μm, >1 μm, >2.5 μm, >5 μm, and >10 μm. The sensors use an unknown algorithm to convert NC to PM_1_ (particles with aerodynamic diameter <1 μm), PM_2.5_ (particles with aerodynamic diameter <2.5 μm), and PM_10_ (particles with aerodynamic diameter <10 μm) mass concentration. Finally, PAir is also equipped with a BME 280 sensor (Bosch Sensortec GmbH, Reutlingen, Germany) to record temperature and relative humidity. PAir wirelessly transmits 2 min averaged data using a WIFI module. Table 1 shows the specifications of PAir particle monitor. 

#### 2.1.1. Measurement Setup in Patras

**Figure 1:**
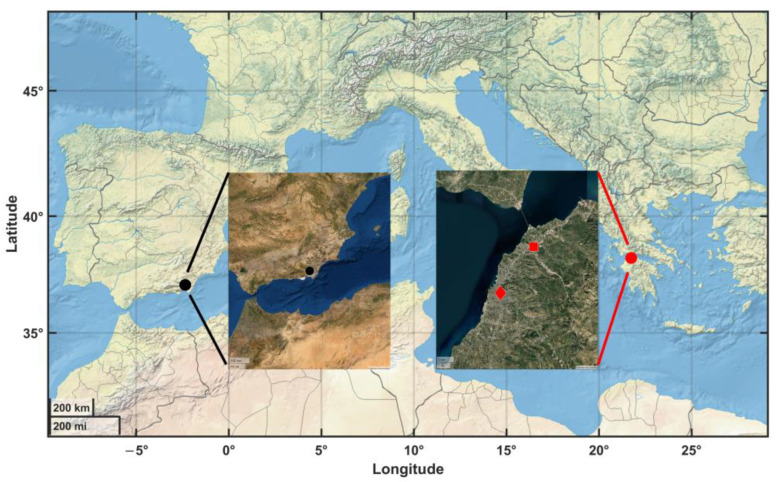
Map of the study sites at Almería, Spain (black circle) and Patras, Greece (red circle). The inset maps zoom in the Almería (PSA), Patras urban (Germanou, red diamond), and Patras background (UPat, red square) experimental sites.

Two sampling sites (urban and background) were in Patras, Greece (Figure 1). Patras is in southern Greece, the third biggest city, with approximately 220,000 inhabitants. During the experimental campaign, a reference instrument was collocated along with the PAir sensors in two areas across the city with different characteristics. The first site was a background one (UPat, 38.29° Ν, 21.78° Ε, 44.5 m asl), ~10 km north of the city center, with limited local PM sources and vehicular air pollution. Air quality conditions remain low and rather stable during the year, mostly affected by regionally transported particles [4]. The second measurement campaign was set up in an urban site (Germanou, 38.24° Ν, 21.74° Ε, 55 m asl). The site is near the city center and major city roads. Air pollution conditions are because of the high traffic density and residential wood-burning emissions for residential heating, especially in winter [4,33]. Both sites are affected by regional and transboundary sources, such as Sahara dust events that occur mostly during spring and autumn in Greece [34,35].

The reference measurements were conducted using a Grimm analyzer (GRIMM Environmental Dust Monitor 180, EDM 180). EDM 180 exploits light-scattering properties to determine PM_1_, PM_2.5_, and PM_10_ mass concentrations. Particles scatter light produced from a diode laser (660 nm), which is captured by a detector. The signal is assigned to 31 size channels (0.25–32 μm) and then converted to mass concentration with 0.1 μg m^−3^ resolution. The particle analyzer was operated according to the manufacturer’s standards and was used to evaluate the PAir size distribution data.

The version of this device outputs data fields for particle mass and NC. PM_1_, PM_2.5_, and PM_10_ mass concentrations were recorded along with the NC at 31 size bins, at 1 min temporal resolution. EDM 180 was also equipped with an integrated NAVION membrane to remove humidity. The GRIMM EDM 180 follows the European standard EN 12341, which describes a standard gravimetric method for determining PM_10_ or PM_2.5_ mass concentrations of suspended particulate matter in ambient air by sampling the particulate matter in ambient air. In this study, the measurements were averaged in hourly resolution. 

In Patras, measurements took place from 24 December 2020 to 16 April 2021 and from 28 January 2022 to 7 April 2022 for the Germanou and UPat sites, respectively. Both sites showed data completeness higher than 93% for the hourly averaged data during the experimental campaigns (Table 2).

#### 2.1.2. Measurement Setup in Almería

The second experimental campaign was performed at CIEMAT’s Plataforma Solar de Almería (PSA) in southern Spain (37.09° Ν, 2.35° W, 495 m asl). The experimental setup was composed of a PAir sensor collocated with a Grimm spectrometer at the KONTAS meteo Grimm EDM164 particle counter, which provided measurements of the particle size distribution at PSA. PSA is located in the desert of Tabernas next to a country road that connects the village of Tabernas (4 km SW) with other villages. The EDM 164 was mounted about 800 m from the road at 2 m height. The surrounding area is partly used for agriculture (almond and olive trees, pasture) and photovoltaic plants, and there is a gypsum mine 5.5 km SSE from PSA. The analyzer shares the same operation protocols as that installed on the Patras site, and the specifications are shown in Table 1. Moreover, the collection efficiency of the EDM 164 device decreases with the particle size. The collection efficiency describes the proportion of particles of a certain size that reach the measuring chamber of the device, relative to the proportion of particles dissolved in the outside air. Part of the particles cannot follow the airflow to the measurement chamber due to inertia, depending on their diameter, mass density, and curve. The collection efficiency of the EDM 164 is only 20% for particles with a diameter of 20 µm and above according to a personal communication with the manufacturer. The difference between the two reference instruments is that the PSA device only outputs particle NC in 31 size bins (0.25–32 μm, as in Patras). The PM mass concentrations were not provided, and thus should be calculated. The PMs calculations assume the spherically shaped particles with a mean particle density (*ρ*) of 2.65 g cm^−3^. This value of particle density is derived as an average of density measurements for mineral dust from Wagner et al., 2009 [36]. The mass concentration at the desired size *bin i*, *m_bin,i_* (in μg), is calculated through Equation (1).

(1) $$m_{bin,i}=\left({NC}_{i}-{NC}_{i+1}\right){\rho V}_{bin,i}$$

where *NC_i_* and *NC_i+_*_1_ are the number concentrations (in # particles cm^−3^) of all particles at the size bins *i* and *i* + 1, *ρ* is the particle’s density, and *V_i_* is the volume of the particles (in cm^−3^) in the *i*-th size channel. Since all particles are considered to have spherical shapes, the volume in *i*-th size channel (*V_bin,i_*) is derived as:

(2) $$V_{bin,i}=\frac{{\pi d}^{3}}{6}$$

with *d* mean diameter of the upper (*I* + 1) and the lower (*i*) channel diameter. The desired PM fraction is calculated by aggregating the appropriate mass bins.

The measurements were conducted from 16 December 2019 to 9 January 2022, including days with varied weather and conditions, and hourly averaged data reported a data completeness of 73% (Table 2). The missing data from 11 August 2021 to 20 October 2021 are because of the annual maintenance and calibration of the Grimm analyzer.

### 2.2. Data Quality Assurance and Sensor Precision

FEM monitoring of data accuracy is evaluated through annual calibration and maintenance processes. Thus, Grimm data are considered to meet all the regulatory and precision protocols. However, several NC data were accompanied by erroneous meteorological data (temperature and RH) from the integrated sensors. This is because of datalogger data acquisition anomalies. All these records were flagged as erroneous and omitted from the analysis. The device outputted data in 1 min temporal resolution and then were averaged in 1 h time intervals.

On the other hand, LCSs are often prone to non-robust observations, and more detailed data cleaning is necessary [7]. As mentioned previously, PAir measurements from the two integrated PMS 5003 sensors had a 2 min temporal resolution, and in this case, it was vital to ensure the intra-unit precision of the identical sensors in each PAir package. The manufacturer provided instructions on data quality assurance. Data should be deleted when the PM_2.5_ concentration is lower than 100 μg m^−3^ and the difference between sensors A and B is higher than 10 μg m^−3^. Similarly, for PM_2.5_ concentrations higher than 100 μg m^−3^, only data with a percentage difference lower than 10% were valid. Also, if one of the two sensors reported missing data or abnormal temperature or humidity conditions, data were omitted. 

A method proposed by Kosmopoulos et al. [18] was also implemented, providing extra quality control. Data with PM_1_ (or PM_2.5_) difference between the two sensors measurements less than 20% of their average or less than 2 μg m^−3^ were considered valid. The second constraint was required to avoid the exclusion of substantially low concentrations. The limit of 2 μg m^−3^ was estimated as the upper fence of the measured concentration differences between the two channels. Minimum data completeness of 70% was also required for the hourly averages to be representative. Figures S1–S3 represent the agreement of Sensors A and B, while the coefficient of variation (*CV*), which is an indicator of duplicate units measurements reproducibility and agreement, was also calculated based on the hourly measurements of the identical PMS 5003 sensors as follows:

(3) $$CV=100\times \frac{\sigma }{\mu }$$

where *σ* is the standard deviation and *μ* is the mean value of the PAir measurements. 

The average CVs (%) for each PAir sensor were 8.3 ± 7.6%, 2.1 ± 1.9%, and 3.4 ± 3.6% at the Almería, Germanou, and UPat sites, providing good reproducibility. The average of the duplicate PMS 5003 sensor readings in each PAir module is kept in the forthcoming analysis.

## 3. Results

### 3.1. Grimm Particle Number Size Distributions

The averaged number size distribution of particles ranged from 0.25 to 32 μm (compared to the range of 0.3–10 μm for PAir), measured by the Grimm analyzer in 31 discrete channels at the examined areas. The Grimm size distribution suggests the different atmospheric profiles of the different locations (Figure S4). The areas shared a similar size distribution shape with a decreasing number of particles for larger particles, but their profile became rather distinct for particles > 2.5 μm. For smaller particles (0.3–2.5 μm), NC_0.3–2.5_ in Germanou were 158% and 156% higher than those reported in PSA and Upat. On the contrary, Almería’s semi-arid area showed approximately 32% and 11% higher concentrations than Germanou and Upat for larger particles (2.5–32 μm). This was expected, since PSA is an area strongly affected by coarse particles originating from local sources or transported dust particles from the Sahara. It has been reported that during dust outbreaks, particle concentrations exceed the thresholds of the World Health Organization (WHO) or EU [37,38,39]. Consequently, investigation of LCS response under conditions where coarse particles are dominant would facilitate and enhance their extensive application. 

Aerosol particles in the other two sites (Germanou and Upat) mainly originate from anthropogenic activities, such as vehicle circulation and residential heating emissions (biomass burning), as well as regional sources. Sea salt, sulfates, and mineral dust have been identified as the main regional sources significantly deteriorating local air quality [40,41]. Moreover, Dimitriou et al. [42] reported that long-range transported particle intrusion is mainly attributed to north-east airflows. In these areas, Sahara dust intrusion events occur only occasionally during late autumn and springtime [43,44]. Thus, the deployment and evaluation of LCSs in regions with different types and origins of aerosol particles could provide more comprehensive information regarding their performance under various atmospheric conditions.

### 3.2. Daily Number Concentrations

**Figure 2:**
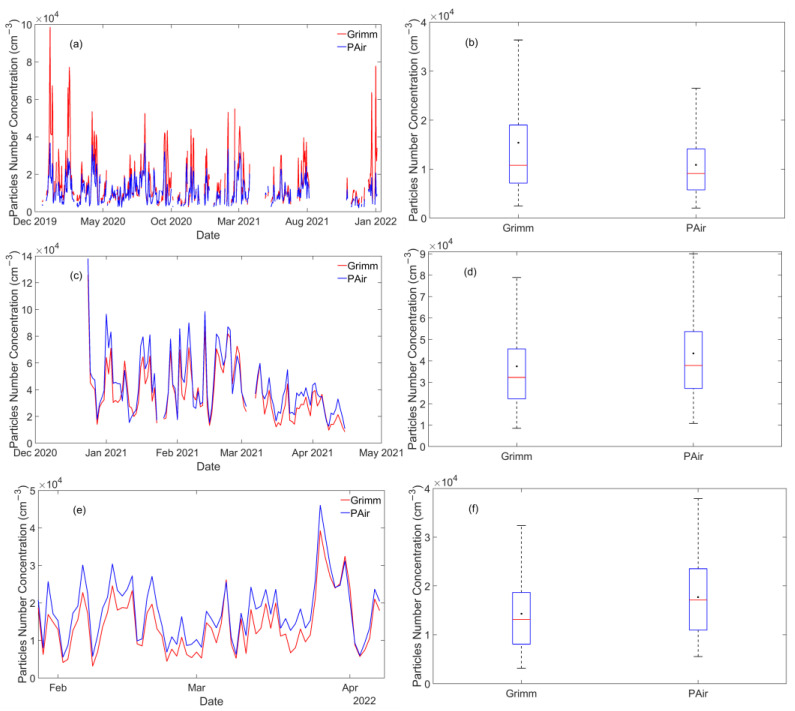
Daily Number Concentrations for particles 0.3–10 μm measured by Grimm and PAir in PSA (**a**), Germanou (**c**), and UPat (**e**). The right panel depicts the boxplots of daily average particle number concentrations in PSA (**b**), Germanou (**d**), and UPat (**f**). The black dots and the solid red lines correspond to the mean and median number concentrations. The bottom and top edges of the box indicate the 25th and 75th percentiles.

Figure 2 represents the daily averaged concentrations for the PAir sensors and Grimm analyzer in the examined areas. Since PAir measurements range between 0.3 and 10 μm, only particles with diameters within these limits were examined so that both instruments shared the same bin widths. For all areas, the LCSs readings, shown in blue (Figure 2), followed a similar daily pattern as the reference instruments, adequately capturing the daily fluctuations of particle NC. PAir was in good agreement with Grimm, reporting R^2^ = 0.69 in PSA. The correlation of determination was higher in Germanou (R^2^ = 0.9) and UPat (R^2^ = 0.92). These findings suggest that the sensor’s behavior differs among the experimental sites (and the different measurement periods), probably due to the variation of ambient particle sizes and distributions.

The experiment in PSA shows that the PAir sensor constantly underestimated ambient particles (Figure 2a,b). The LCS output was 30% lower than Grimm. The average daily NC, along with the standard deviation, were 15,389 ± 12,910 cm^–3^ (2483–98,455 cm^–3^) and 10,878 ± 6728 cm^–3^ (2063–36,708 cm^–3^) for Grimm and PAir, respectively. Hence, the PAir sensor does not appear able to accurately detect particles in this semi-arid area. Moreover, the systematic bias (mean bias error, MBE) was –192 cm^–3^, reporting LCSs’ lower response to coarse particles. The particle accumulation during periods rich in coarse particles could affect the sensor’s sampling. Due to inertial deposition, larger particles’ flow through the sampling area was hampered, provoking losses of larger particles on the walls [7,26]. Consequently, PAir sensors underestimated the actual particles’ NC and their readings were up to ~5 times lower than the reference ones. In general, higher biases are reported when the sensors are implemented in areas where particle size distribution is not stable when compared to controlled environments [24,25,26,45]. Thus, dust events may provoke the observed differences in PSA.

The PAir sensor’s response was altered in the Patras urban (Figure 2c,d) and background sites (Figure 2e,f). In both sites, LCS readings were higher than the reference instrument. In Germanou, PAir reported an average value of 43,443 ± 22,511 cm^–3^, ~16% higher than Grimm (37,419 ± 20,272 cm^–3^), with MBE equal to 259 cm^–3^. Similar results were reported in UPat, where the NC for PAir and Grimm were 17,706 ± 80,303 cm^–3^ and 14,296 ± 76,084 cm^–3^, and the relevant percentage difference was 21%. MBE was lower than the one reported in Germanou and equal to 146 cm^–3^, mainly due to the decreased particle size in this area, as will be discussed in the following sections.

In order to verify the statistical difference of mean among Grimm and PAir, a two-sample t-test was conducted at the 95% confidence level. The t-test showed significant differences among the average NC with *p*-value < 0.05.

The discrepancies in sensor performance among areas with different aerosol characteristics suggests that their sensitivity is affected by particle size and composition. To better investigate that effect, in the forthcoming analysis, several size bins (0.3–0.5 μm, 0.5–1 μm, 1–2.5 μm, 2.5–5 μm, and 5–10 μm) will be examined separately.

### 3.3. Comparison of Pair and Grimm Particle Number Concentration Measurements

The examined areas have distinct air quality characteristics, as discussed in previous sections. Thus, differences are expected in the average particle NC. In order to investigate the discrepancies between PAir and Grimm outputs, we investigated NC in 3 size bin channels, for particles with the diameters: 0.3–1 μm (NC_0.3–1_), 1–2.5 μm (NC_1–2.5_), 2.5–10 μm (NC_2.5–10_) in the Patras sites. In Almería, two additional size bins were calculated, 2.5–5 μm (NC_2.5–5_) and 5–10 μm (NC_5–10_), to better access LCSs response when coarse particles were dominant.

#### 3.3.1. Germanou Urban Site

**Figure 3:**
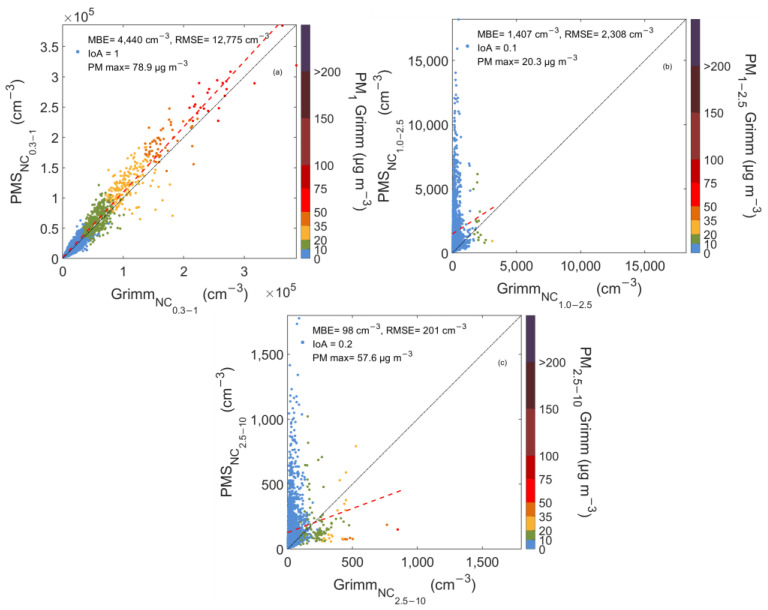
Scatterplots between PAir and Grimm number concentration measurements for particles with diameters 0.3–1 (**a**), 1–2.5 (**b**), and 2.5–10 μm (**c**) in Germanou Urban Site. The color bar shows the corresponding mass concentrations, PM_1_ (**a**), PM_1–2.5_ (**b**), and PM_2.5–10_ (**c**) as reported by Grimm. The 1:1 and the regression lines are also shown in black and red dotted line respectively.

The scatterplots of Figure 3 represent the relationship between the hourly average NC of PAir and Grimm at three size bins (0.3–1 μm, 1–2.5 μm and 2.5–10 μm) in Germanou from December 2020 to April 2021. The sensor showed different responses for the various size bins, in agreement with Zamora et al.’s [23] findings. The normalized mean bias error (NMBE), MBE divided by the average value of the reference instrument, was calculated to investigate the discrepancies between the instruments’ measurements. The calculated NMBE values were 12%, 629%, and 212% for particles with diameters of 0.3–1, 1–2.5, and 2.5–10 μm, respectively, showing a different sensor response for varying particle sizes.

In order to detect the discrepancies between the LCSs and the reference instrument, the Index of Agreement (IoA) of the hourly averaged values, as proposed by Willmott (1981), was calculated. IoA represents the ratio of the mean square error and the potential error, with values close to 1 indicating a perfect match, while values close to 0 indicate that there is no agreement between the two instrument measurements [46].

The size range of 0.3–1 μm (Figure 3a) showed a linear response and an excellent agreement between PAir and Grimm (IoA = 1). PAir average NC_0.3–1_ was 41,302 ± 44,855 cm^−3^, 12% higher than Grimm (36,861 ± 40,146 cm^−3^). The color bar in Figure 3a illustrates the PM_1_ concentrations reported by the reference instrument. During the measurement campaign, PM_1_ was at low levels, ranging from 0.5 to 79 μg m^−3^ and an average of 9.2 ± 9.4 μg m^−3^. In this urban site, PM_1_ range did not show any significant effect on the sensors’ size distribution sensitivity. This fact explains that, for the fine fraction mode and during periods with good air quality conditions, LCSs adequately reproduced the particle’s NC.

Figure 3b depicts the sensors’ performance for the channel bin corresponding to particles with a diameter of 1–2.5 μm. Pair sensors do not report that size bin, but it was calculated by subtracting the channels referring to particles with diameters > 1 μm and >2.5 μm. Compared to the previous size bin (0.3–1 μm), a completely different behavior is depicted, with LCSs consistently overestimating particle NC. While Grimm suggested that a small portion of particles lay in the 1–2.5 μm size mode (PM_1–2.5_ = 1.3 ± 1.6 μg m^−3^), PAir significantly overestimated particle concentrations within that range. Grimm’s NC data ranged from 15 to 3111 cm^−3^, while the LCSs records were one order of magnitude higher, reaching 18,193 cm^−3^. Despite this overestimation, the calculated slope was lower than 1 (0.65). Previous works have also reported these limitations on sensor responses for larger particles [26]. However, these results are not aligned with the results reported by Li et al. [29]. Experiments conducted under controlled conditions showed that PAir underestimated the NC for various particle types, demonstrating the possible particle sources and composition effect on sensor precision. These discrepancies demonstrate the need to conduct a more precise and size-bin-specific calibration procedure.

Similar results were extracted for the 2.5–10 μm size bin. NC derived from PAir did not agree with Grimm (Figure 3c), suggesting that this bin was also noisy. While coarse particle concentrations remained low in that site (average mass concentration was PM_2.5–10_ = 3 ± 4.1 μg m^−3^, ranging between 0.1–57.6 μg m^−3^), PAir NC shifted towards larger particles, assigning a significant number of particles in this size bin. The LCSs’ mean NC was 144 ± 172 cm^−3^, while Grimm reported only 46 ± 62 cm^−3^. The higher standard deviation (172 cm^−3^) reported from PAir indicates an increased fluctuation compared to the real ambient conditions (62 cm^−3^).

An interesting finding is that sensor behavior changes when PM_2.5–10_ mass concentrations exceed 10 μg m^−3^. Data points appear to follow a line with a different slope compared to the rest of the measurements, underestimating NC. This suggests that sensor response changes during periods with increased levels of coarse particles, and that size generally affects sensors performance. 

#### 3.3.2. UPat Background Site

**Figure 4:**
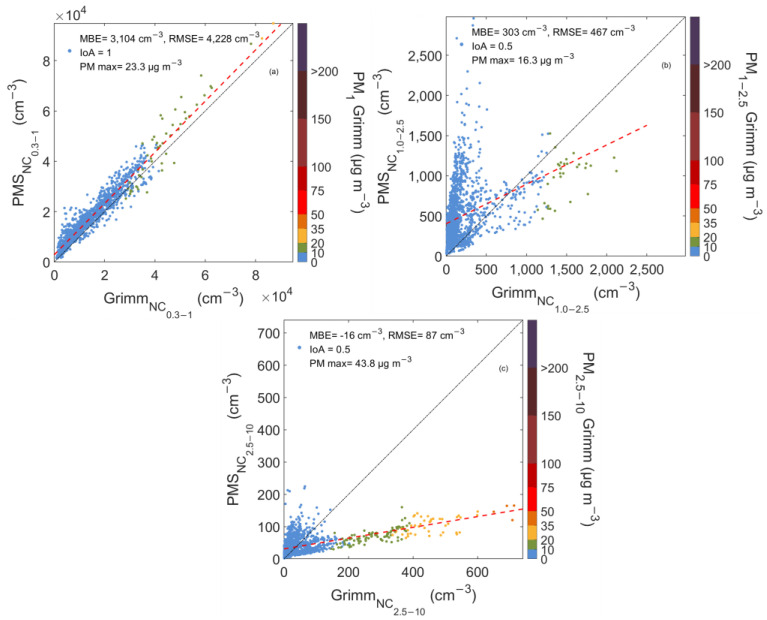
Scatterplots between PAir and Grimm number concentration measurements for particles with diameters 0.3–1 (**a**), 1–2.5 (**b**), and 2.5–10 μm (**c**) in UPat Background Site. The color bar shows the corresponding mass concentrations, PM_1_ (**a**), PM_1–2.5_ (**b**), and PM_2.5–10_ (**c**) as reported by Grimm. The 1:1 and the regression lines are also shown in black and red dotted line respectively.

The second experimental setup was also conducted in the greater area of Patras, Greece in the UPat site. Figure 4 shows the relationship between PAir and Grimm for the three different size bins. The regression analysis results for the first channel bin (0.3–1 μm) were similar to those reported in the urban site, showing a significant variation among the examined bins’ slope (0.17–1.02) and NMBE 22% for NC_0.3–1_, 153% for NC_1–2.5_, and −28% for NC_2.5–10_. 

During the campaign, Grimm NC_0.3–1_ average value was 14,078 ± 10,292 cm^−3^, PM_1_ concentrations ranged from 0.3 to 23 μg m^−3^, and the sensor showed an excellent reproducibility of ambient data with an average NC_0.3–1_ of 17,182 ± 10,857 cm^−3^. The sensor’s response was linear, exhibiting IoA and slope values close to unity, 1 and 1.02. PM_1_ was low, with an average value of 4 μg m^−3^ recorded by the reference unit, and 97% of the data were lower than 10 μg m^−3^. This suggests that PAir response to particles in the size bin of 0.3–1 μm is satisfactory during good air quality conditions.

Figure 4b depicts the scatter plot between Pair and the Grimm for the second size bin (1–2.5 μm). PAir showed poor agreement with Grimm (IoA = 0.5), with two branches observed in the graphs. The first branch (denoted with blue) indicates that LCSs clearly overestimated the NC for particles with aerodynamic diameter from 1 to 2.5 μm during periods when PM_1–2.5_ mass concentration is lower than 10 μg m^−3^. When PM_1–2.5_ concentrations exceeded 10 μg m^−3^, LCSs underestimated the NC and most data points were located under the 1:1 line (black dashed line). Thus, for increasing particle sizes, sensor sensitivity seems to worsen, and this behavior requires further investigation.

PAir sensors cannot accurately detect coarser particles, as illustrated in Figure 4c. The presence of an increased number of coarse particle concentrations appears to affect sensors’ NC response strongly. A total of 136 hourly cases with PM_2.5–10_ concentrations higher than 10 μg m^−3^ were identified, with 95% of them observed from 28/03 to 07/04. For this period, the method proposed by Kosmopoulos et al. [18] was applied to identify possible dust events. More specifically, the coarse-to-fine-particle fraction was calculated, PM_2.5–10_/PM_2.5_, using the hourly averaged measurements. If the fraction exceeded the threshold value of 0.43, coarse particles dominated. During that period, coarse particles were dominant, with an average value of PM_2.5–10_ = 11 μg m^−3^ and PM_10_ = 23.5 μg m^−3^, while for the rest of the dataset, the average values were 5.6 and 4.4 μg m^−3^, respectively.

These dust events provide valuable information on sensors’ performance in background sites when coarse particles dominate. LCS’s response was poor, and its observations drifted towards lower NC. During that period, NC_2.5–10_ was 83 ± 29 cm^−3^ and 344 ± 119 cm^−3^ for PAir and Grimm, respectively, suggesting a difference of 121% between the two instruments’ measurements. This behavior during coarse particle events has been reported in previous studies [4,19,20], mainly attributed to sensor structure [9].

#### 3.3.3. PSA Semi-Arid Area

**Figure 5:**
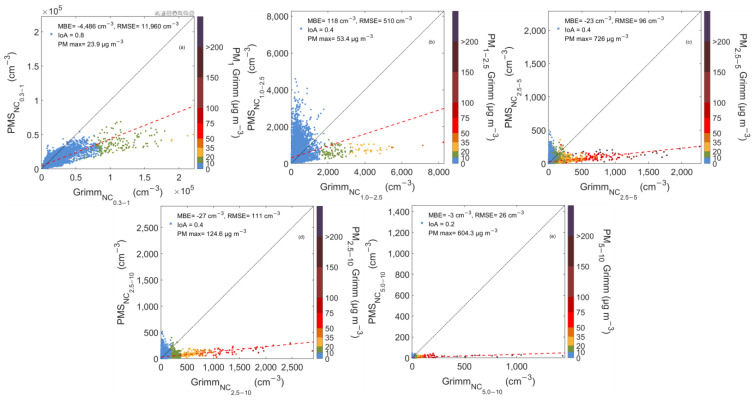
Scatterplots between PAir and Grimm number concentration measurements for particles with diameters 0.3–1 (**a**), 1–2.5 (**b**), 2.5–5 (**c**), 2.5–10 (**d**), and 5–10 μm (**e**) in PSA semi-arid area. The color bar shows the corresponding mass concentrations, PM_1_ (**a**), PM_1–2.5_ (**b**), PM_2.5–5_ (**c**), PM_2.5–10_ (**d**), and PM_5–10_ (**e**), as reported by Grimm. The 1:1 and the regression lines are also shown in black and red dotted line respectively.

A semi-arid area in Almería, Southern Spain was selected to better investigate the effect of coarse particles. Figure 5 shows the scatter plot of the low-cost sensors against the reference measurements.

Although PM_1_ concentrations ranged between similar levels, from 0.1 to 24 μg m^−3^, similar to Patras, the linear regression equations suggest a distinct behavior. The sensor evaluation was conducted over a wide range of NC_0.3–1_ (870–222,257 cm^−3^), as reported by Grimm. Most data points (77%) are located beneath the 1:1 line. The linear regression slope was 0.39, and the NMBE was −31%. These findings suggest a clear underestimation of particle NC. The sensor’s operation protocols and particles composition could be the reason for its limited sensitivity at the examined area, even for particles smaller than 1 μm.

The sensor’s response pattern was also similar for the second and third of the examined bins that corresponded to particles of aerodynamic diameter ranging between 1–2.5 μm and 2.5–10 μm, respectively. The results suggest once again the LCS’s inability to adequately record the NC of particles with a diameter higher than 1 μm. The calculated IoA value was 0.4 for both NC_1–2.5_ and NC_2.5–10_. PAir outputs for both size bins did not agree with Grimm, raising several concerns about the sensor’s utility across areas where coarse particles are dominant. The two instruments’ readings differed by 33 and 55% for NC_1–2.5_ (NMBE = 41%) and NC_2.5–10_ (NMBE = −43%). This behavior is expected, considering the experimental site’s characteristics: PM_1–2.5_ and PM_2.5–10_ concentrations ranged between 0.1 and 53 μg m^−3^ and 0.9–124 μg m^−3^, respectively, so several cases with elevated coarse particles concentrations were reported.

To better assess the PAir sensor’s performance on coarse particles measurements in PSA, additional size fractions were investigated (2.5–5 μm and 5–10 μm). Approximately 15% of the measurements reported PM_2.5–5_ mass concentrations higher than 10 μg m^−3^. These data points corresponded to NC_2.5–5_, as reported by the reference instrument, varying from 33 to 2263 cm^−3^ with an average value of 183 ± 204 cm^−3^. The corresponding reading from the LCS ranged from 5 to 360 cm^−3^ with a mean value of 41 ± 43 cm^−3^ and NMBE = −42%. The measurement biases indicate the necessity of an appropriate PAir calibration across semi-arid areas, especially during days when coarse particles are dominant.

Finally, ambient Grimm NC for particles between 5 and 10 μm reached up to 1440 cm^−3^, but the PAir sensor could not measure data sufficiently. An underestimation of reference measurements (NBME = −43%) is shown with an average value of 4 cm^−3^ (1–42 cm^−3^), a decrease of 55% decrease compared to Grimm. During the experimental period, 274 cases when PM_5–10_ mass concentrations exceeded 10 μg m^−3^ were recorded. All the corresponding data points lie under the 1:1 line, since the PAir sensor significantly underestimated ambient conditions. While the Grimm analyzer reported an average PM_5–10_ concentration of ~30.3 μg m^−3^ and NC_5–10_ of 148 cm^−3^, the low-cost unit measurement was decreased by ~90% (14 cm^−3^). On the other hand, when PM_5–10_ mass concentrations were examined, the discrepancies between the low cost and the reference units were smaller, with the PAir sensor underestimating Grimm by approximately 50%.

In general, LCSs discrepancies shown in PSA are in accordance with previous works in environments and periods rich in coarse particles [24], findings that underline the particle size effect on sensor performance. The sensor’s precision when measuring particles > 1 μm is highly related to particle size distribution. For environments where number and mass concentrations fluctuate in a narrower range, LCSs can provide reasonable results. In contrast, in areas where particles >1 μm are dominant (e.g., PSA), sensors are relatively insensitive to particle fluctuations [24].

### 3.4. PAir Performance When Coarse Particles Are Dominant in PSA

**Figure 6:**
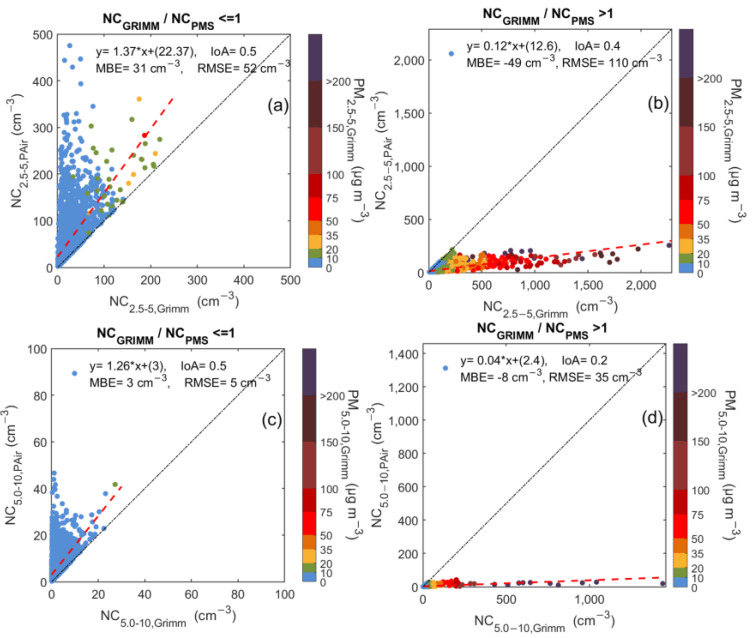
Scatterplots between PAir and Grimm number concentration measurements for particles with diameters 2.5–5 (**a**,**b**) and 5–10 μm (**c**,**d**) during the period when the fraction of Grimm measurements to PAir ones is lower than 1 (left panel) and higher than 1 (right panel) in the PSA semi-arid area. The color bar represents the corresponding mass concentrations PM_2.5–5_ (**a**,**b**) and PM_5–10_ (**c**,**d**), as reported by Grimm. The 1:1 and the regression lines are also shown in black and red dotted line respectively.

To better investigate the effect of coarse particles on LCS performance in PSA, the dataset was split into two subsets, namely N_GRIMM_/N_PMS_ ≤ 1 and N_GRIMM_/N_PMS_ > 1. This method was applied to the size bins of 2.5–5 μm and 5–10 μm. The regression analysis results for these two subsets are shown in Figure 6. When the NC of particles with diameter 2.5–10 μm was examined, 32% of hourly measurements had N_GRIMM_/N_PMS_ ≤ 1 (Case A), while 68% had N_GRIMM_/N_PMS_ > 1 (Case B). The respective percentages for particles 5–10 μm were 44% (Case C) and 56% (Case D). Comparison between the time periods with fractions higher and lower than unity showed that different atmospheric conditions were dominant. During cases A and C, Grimm measurements indicate that particle concentrations remained low. NC were lower than 200 for NC_2.5–10_ and 25 cm^−3^ for NC_5–10_, while PM_2.5–5_ and PM_5–10_ averages were 1.8 ± 2.43 μg m^−3^ and 0.69 ± 0.79 μg m^−3^, respectively. These values indicate that during these periods, particle levels remained very low with minimal hourly deviations. The PAir observations significantly overestimated the ambient conditions reporting slopes of 1.37 and 1.26 for the two size bins.

PAir NC measurements during cases B and D showed a different response. Figure 6b,d show the relationship of the LCSs’ hourly measurements with the reference instrument. While Grimm reports number concentrations reaching up to 2200 cm^−3^ for NC_2.5–5_ and 2000 cm^−3^ for NC_5–10_, the LCS seems insensitive to these elevated values, and the highest number concentrations were 400 and 250 cm^−3^ for NC_2.5–5_ and NC_5–10_. This alteration in sensors behavior is attributed to particle type and size. 

PM_2.5–5_ and PM_5–10_ concentrations were roughly four and seven times higher than those during periods A and C, confirming again the inability of PAir sensors to capture the observed air quality conditions as coarse particles dominated.

### 3.5. Dust Events 

**Figure 7:**
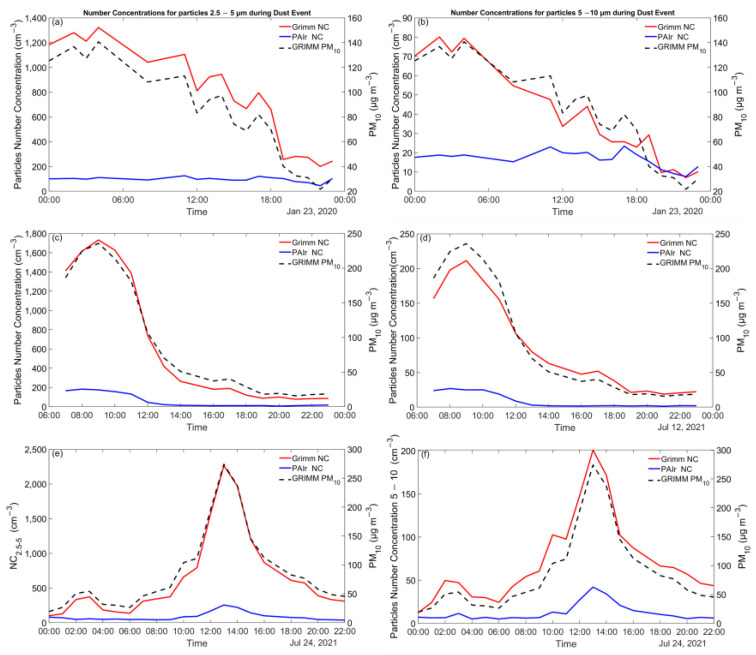
Daily PM_10_, NC_2.5–5_ (left panel), and NC_5–10_ (right panel) concentrations during dust events on 23 January 2020 (**a**,**b**), 12 July 2021 (**c**,**d**), and 24 July 2021 (**e**,**f**) in PSA as reported by Grimm (red and black dashed line) and Pair (blue line).

Dust intrusion episodes usually affect arid and semi-arid areas. Southern regions of the Iberian Peninsula are prone to dust events from local sources or long-range transported particles from the Sahara [47,48,49]. During periods B and D, three dust events occurred, provoking elevated PM_10_ mass concentrations. These events took place on 23 January 2020, 12 July 2021, and 24 July 2021. The dust events were also identified by Multiscale Online Nonhydrostatic AtmospheRe CHemistry model (MONARCH) products [50,51], developed by Barcelona Supercomputing Center (BSC), that provides valuable products concerning mineral dust and aerosols and the observations of the satellite Moderate Resolution Imaging Spectroradiometer (MODIS). Figure 7 shows the hourly time series for the number and mass concentrations reported by GRIMM and PAir. Two size bins were examined: 2.5–5 μm and 5–10 μm. Table 3 includes the daily statistics for these days.

During the dust events, PM_10_ daily averaged data ranged between 82 and 91 μg m^−3^_._ The highest concentration for particles 2.5–5 μm recorded by Grimm was on 23 January 2020, when NC_2.5–5_ was 773 ± 387 cm^−3^ and the corresponding PAir value was 97 ± 18 cm^−3^ (MBE = −677 cm^−3^). The highest value for particles with diameter 5–10 μm was recorded on 12 July 2021 (87 ± 70 cm^−3^), with the LCSs reporting an MBE of −78 cm^−3^ (NC_5–10_ = 9 ± 10). These findings indicate approximately 8 and 10 orders of magnitude difference between the two instruments’ measurements for each fraction. 

## 4. Conclusions

In this study, an evaluation of PAir low-cost sensors’ selectivity on particle number concentrations was conducted in three different ambient environments. The sensors reported an adequate response on the daily NC fluctuations for particles with diameters within 0.3–10 μm (R^2^ was 0.69–0.92). In the case of different size bins, the sensors provided ambiguous results. Particle size and type appeared to affect the performance of the sensors. When deployed in the urban and background sites, PAir sensors accurately reported NC for particles with a diameter of 0.3–1 μm, while a systematic overestimation was observed for coarser particles (>1 μm). Sensor response alternated when the experimental campaign was conducted in a semi-arid area with high coarse particle concentrations. Number concentrations from PAir were lower than Grimm and significant biases were reported. The decreased response for larger particles could be attributed to the sensors sampling path structure that provokes particle deposition losses. Finally, three dust events were identified during the measurement period in the semi-arid area. Pair evaluation showed that the sensor is rather insensitive to measuring NC during periods when coarse particles are dominant and failed to accurately represent NC for particles 2.5–5 and 5–10 μm.

LCSs’ dense deployment can provide valuable information for air quality conditions. Since their measurement quality is still challenging, thorough evaluations under various environments are necessary. Assessment of their NC outputs could allow a better understanding of their performance and precision. Moreover, an appropriate size-specific calibration procedure for each size bin could provide more reasonable estimates of particle concentrations. 

## Figures and Tables

**Table 1:** Manufacturer technical data for Pair and Grimm instruments located in UPat (38.29° Ν, 21.78° Ε), Germanou (38.24° Ν, 21.74° Ε) (Grimm EDM 180 and PAir), and PSA (37.09° Ν, 2.35° W) (Grimm EDM 164 and PAir).

Parameter	Grimm EDM 180	Grimm EDM 164	PAir
Size Channels Output	0.25–32 μm (in 31 channels)	0.25–32 μm (in 31 channels)	>0.3 μm, >0.5 μm, >1 μm, >2.5 μm, >5 μm, >10 μm
Mass Concentration Fractions	PM_1_, PM_2.5_, PM_10_ and TSP	PM_1_, PM_2.5_, PM_10_ and TSP	PM_1_, PM_2.5_, PM_10_
Effective Range of Measurements	0.1–1500 μg m^−3^	0.1 to >6000 μg m^−3^	0–500 μg m^–3^
Flow Rate	1.2 ± 5% l min^–1^	Same as EDM 180	0.1 l min^–1^
Light Source Wavelength	685 nm	655 nm	~680 ± 10 nm *
Operational Temperature Range	−20–50 °C	−25–50 °C	−10–60 °C

* The laser wavelength was measured by Sayahi et al. [7].

**Table 2:** Site description.

Station	City	Station Type	Measurement Period	Hourly Data Completeness (%)
PSA	Tabernas	Semi-arid	16 December 2019–9 January 2022	73
UPat	Patras	Background	28 January 2022–7 April 2022	98
Germanou	Patras	Urban	24 December 2020–16 April 2021	93

**Table 3:** PM_10_, NC_2.5–5_, and NC_5–10_ measurement statistics during and dust events.

		PM_10_ (μg m^−3^)		NC_2.5–5_ (cm^−3^)		NC_5–10_ (cm^−3^)	
		Mean ± std	Range	Mean ± std	Range	Mean ± std	Range
23 January 2020	Grimm	82 ± 40	21.6–40.6	773 ± 387	200–1321	38 ± 24	7–80
	PAir	-	-	97 ± 18	44–125	17 ± 4	7–23
12 July 2021	Grimm	91 ± 85	15.7–35.7	632 ± 667	77–1732	87 ± 70	19–211
	PAir	-	-	62 ± 71	6–183	9 ± 10	1–27
24 July 2021	Grimm	86 ± 70	19.4–274	635 ± 601	100–2263	71 ± 49	12–200
	PAir	-	-	85 ± 60	37–255	12 ± 10	5–42

## Data Availability

The data presented in this study are available on request from the corresponding author.

## Supplementary Materials

The following supporting information can be downloaded at: https://www.mdpi.com/article/10.3390/s23146541/s1, Figure S1: Scatterplot of PAir sensors A and B raw PM_2.5_ measurements in PSA.; Figure S2: Scatterplot of PAir sensors A and B raw PM_2.5_ measurements in Germanou; Figure S3: Scatterplot of PAir sensors A and B raw PM_2.5_ measurements in UPat; Figure S4: Average particle size distribution based on Grimm measurements in Almeria, Germanou, and UPat.
