# Supplementary material for: Investigating the Sensitivity of Low-Cost Sensors in Measuring Particle Number Concentrations across Diverse Atmospheric Conditions in Greece and Spain
Source: Sensors (Basel). 2023 Jul 20;23(14):6541. doi: 10.3390/s23146541 (PMC10383866; doi:10.3390/s23146541)
Supplement: Supplementary file 1 [file sensors-23-06541-s001.zip › sensors-2498465-supplementary.pdf]

Supplementary Material

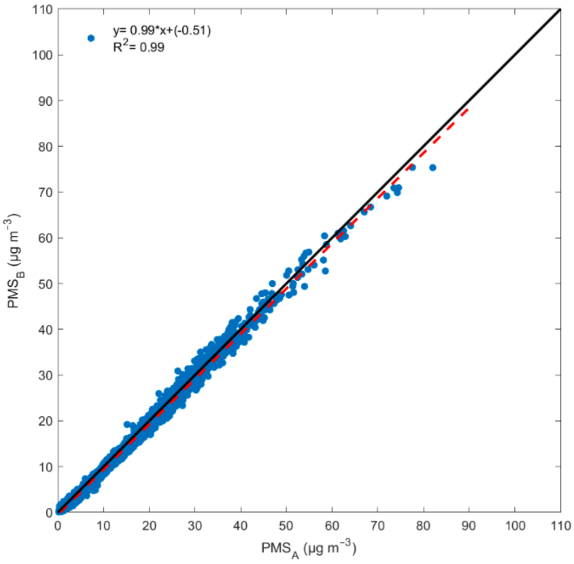

Figure S1. Scatterplot of PAir sensors A and B raw $PM_{2.5}$ measurements in PSA.

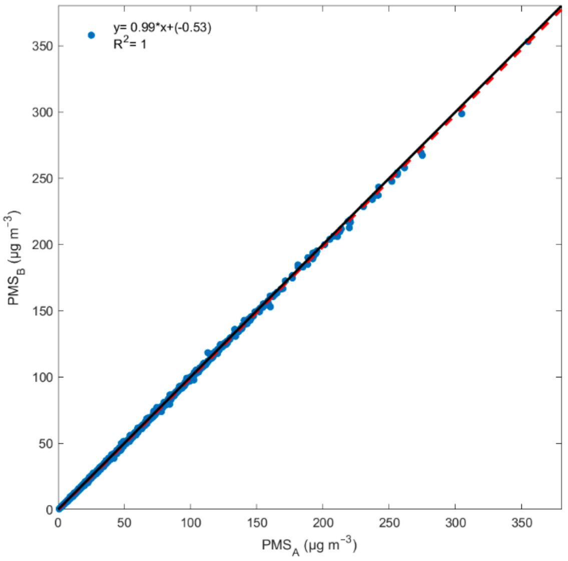

Figure S2. Scatterplot of PAir sensors A and B raw $PM_{2.5}$ measurements in Germanou.

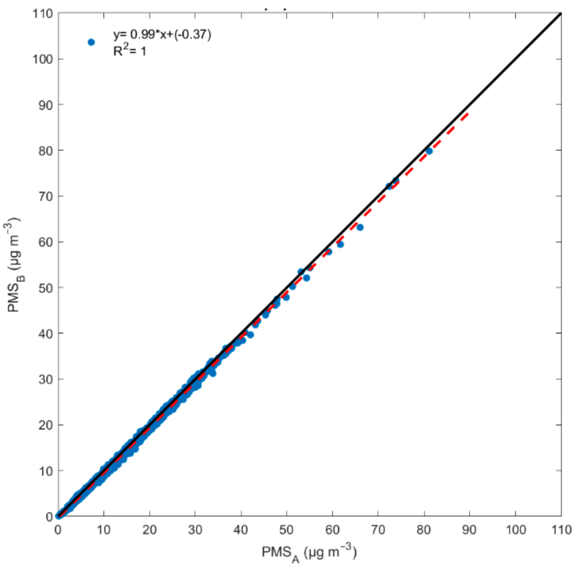

Figure S3. Scatterplot of PAir sensors A and B raw $PM_{2.5}$ measurements in UPat.

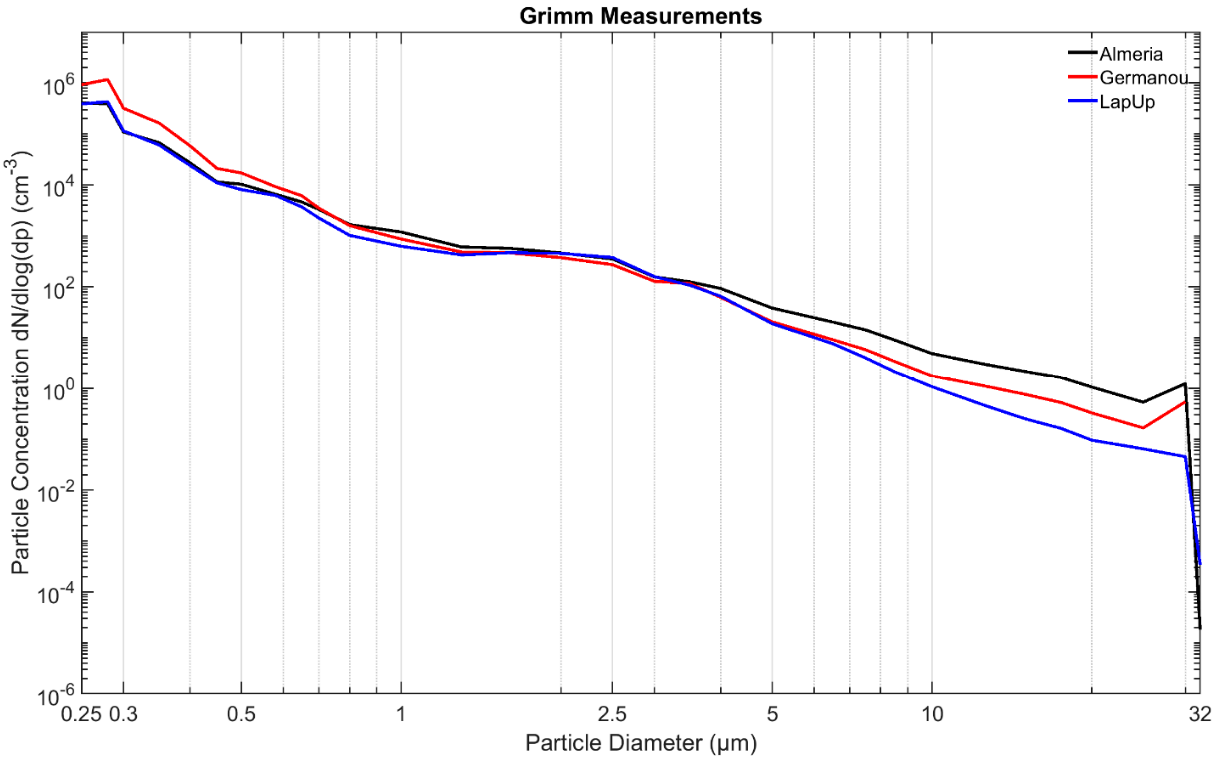

Figure S4. Average Particle Size Distribution based on Grimm measurements in Almeria, Germanou and UPat.
